# Polyextremophilic Chitinolytic Activity by a Marine Strain (IG119) of *Clonostachys* *rosea*

**DOI:** 10.3390/molecules27030688

**Published:** 2022-01-21

**Authors:** Marcella Pasqualetti, Susanna Gorrasi, Valeria Giovannini, Martina Braconcini, Massimiliano Fenice

**Affiliations:** 1 Department of Ecological and Biological Sciences (DEB), University of Tuscia, Largo Università snc, 01100 Viterbo, Italy; mpasqual@unitus.it (M.P.); gorrasi@unitus.it (S.G.); v.giovannini@unitus.it (V.G.); martina.braconcini@unitus.it (M.B.); 2 Laboratory of Ecology of Marine Fungi, CoNISMa, Department of Ecological and Biological Sciences, University of Tuscia, Largo Università snc, 01100 Viterbo, Italy; 3 Laboratory of Applied Marine Microbiology, CoNISMa, Department of Ecological and Biological Sciences, University of Tuscia, Largo Università snc, 01100 Viterbo, Italy

**Keywords:** chitinolytic enzymes, marine fungi, polyextremophiles, high producer, *Clonostachys* *rosea*, response surface methodology

## Abstract

The investigation for novel unique extremozymes is a valuable business for which the marine environment has been overlooked. The marine fungus *Clonostachys rosea* IG119 was tested for growth and chitinolytic enzyme production at different combinations of salinity and pH using response surface methodology. RSM modelling predicted best growth in-between pH 3.0 and 9.0 and at salinity of 0–40‰, and maximum enzyme activity (411.137 IU/L) at pH 6.4 and salinity 0‰; however, quite high production (>390 IU/L) was still predicted at pH 4.5–8.5. The highest growth and activity were obtained, respectively, at pH 4.0 and 8.0, in absence of salt. The crude enzyme was tested at different salinities (0–120‰) and pHs (2.0–13.0). The best activity was achieved at pH 4.0, but it was still high (in-between 3.0 and 12.0) at pH 2.0 and 13.0. Salinity did not affect the activity in all tested conditions. Overall, *C. rosea* IG119 was able to grow and produce chitinolytic enzymes under polyextremophilic conditions, and its crude enzyme solution showed more evident polyextremophilic features. The promising chitinolytic activity of IG119 and the peculiar characteristics of its chitinolytic enzymes could be suitable for several biotechnological applications (i.e., degradation of salty chitin-rich materials and biocontrol of spoiling organisms, possibly solving some relevant environmental issues).

## 1. Introduction

The marine environment is extremely multiform, representing an important source of diversity (biological and chemical) and an enormous reserve of new and/or unexploited microorganisms. Among the marine microbial diversity, a high number of archaea, bacteria and fungi show unique adaptation features to the polyextremophilic conditions imposed by the very harsh environments. For example, in the deep ocean microorganisms are subject to high hydrostatic pressure combined with low or high temperature or to high salinity and alkaline pH. Besides, in various marine transition areas (intertidal zones, estuaries and marine salterns), they are submitted to sudden and repeated variations of temperature and salinity [1,2,3].

Different productive activities (i.e., feed and food, fine chemicals, pharmaceutical, and enzyme industries) could benefit from the ocean’s microbial diversity, which represents a huge source of new substances, including enzymes, with high potential for their specific applications [4,5,6].

The commercial value of enzymes increased in the past 20 years both in traditional fields (i.e., detergent and food industries) and in various diversified sectors, such as bioenergy, biosensors, environmental biotechnology (i.e., depollution), leather/textile industries and in medical applications (where it is still growing) [7,8]. The global enzyme market was valued at $8636.8 million in 2019 and it is projected to reach $14,507.6 million in 2027 [9].

Thus, marine environments should not be disregarded for the search of new enzymes in order to cope with the high needs of this important business, since there is a very broad interest to discover new enzymes for eco-friendly industrial and biotechnological applications [4,10]. New biocatalysts showing unusual properties (tolerance to salt, high and/or low pH and temperature), called extremozymes, can be gained by marine enzyme biotechnology [11,12]. The availability of enzymes, showing their optimal activities at extreme or sub-extreme values of pH, salt concentration, and temperature, is very useful for the technological development. Furthermore, enzymes obtained from marine environments could be particularly interesting for their possible new valuable chemical and catalytical features [5,13]. However, marine environments are still scarcely exploited for new enzymes if compared with terrestrial ones [4,11]. Still inadequate research has been conducted to screen marine microorganisms for their extracellular enzymes production, and marine fungi have been even less investigated [4,14,15]. Among the most studied hydrolytic activities, chitinolytic enzymes still need further investigations for their wide pattern of industrial and environmental applications, where new and suitable sources of biocatalysts are needed [16].

Chitin (homopolymer of *N*-acetyl-β-d-glucosamine with β1-4-linkages) is a very abundant natural polysaccharide, widely diffused both in marine and terrestrial environments, being mainly present in arthropod exoskeleton and fungal cell-wall. Its hydrolysis is carried out by a series of chitin-degrading enzymes (generally referred as chitinases). Even though various innovative applications have been tested at the laboratory scale [17,18], these biocatalysts are generally employed in more traditional fields, such as the hydrolysis of chitin-rich materials, the production of chitin derivatives, the generation of fungal protoplasts, and the biocontrol of pathogenic organisms [16,19,20]. Nevertheless, some of the cited applications (i.e., chitin wastes degradation or pathogen biocontrol), can be strongly restricted by adverse environmental conditions, in particular low temperature, acid/alkaline pH and high salinity, affecting both the microbial growth and the enzymes activity. Here, the search for new microorganisms, able to release enzymes with peculiar or unique characteristics, is useful to solve on-field problems. For example, the screening for new cold-active chitinases by psychrophilic organisms demonstrated to be very useful for applications at low temperature [16,21,22,23]. Also, the search of chitinolytic enzymes, active at high salt concentrations, represents an interesting investigation field with several promising applications. These enzymes can find use in the valorization of chitinous wastes (i.e., shellfish wastes), production of added-value chitin derivatives (i.e., chito-oligosaccharides) under special conditions, preservation of salted food, and biological pest control in xerophilic habitats [24,25,26,27].

However, the search for microorganisms, able to produce high levels of chitinases in halophilic conditions or extreme and/or sub-extreme pH, is quite scarce and mainly regards bacteria [28]. In this context, marine fungi have been somehow overlooked and their ability to grow and produce chitinases in polyextremophilic conditions has never been investigated [29,30,31].

*Clonostachys rosea* IG119 is a new strain, isolated from the marine phanerogam *Posidonia oceanica,* which was preliminary screened for the production of extracellular chitinolytic enzymes, showing a very promising activity [29,32].

In this work, in order to verify *C. rosea* IG119 growth and chitinolytic enzyme production in polyextremophilic conditions, the strain was cultivated under a broad range of pH and salinity. Profiles of growth and enzyme production under the studied conditions (combinations of different pHs and salinities), were obtained by a D-optimal model using the response surface Methodology (RSM). Activity of the crude chitinolytic enzyme solution in polyextremophilic conditions was tested too.

## 2. Results and Discussion

Chitin represents an important source of carbon and nitrogen for marine organisms and its turnover in the aquatic biosphere is quite high. It has been estimated that more than 10^11^ metric tons of this polysaccharide are produced annually and the oceans represent a huge resource of chitin that is transformed in different biological materials to avoid carbon and nitrogen depletion in these oligotrophic environments [33]. Actually, marine sediments contain only traces of chitin, whose degradation is a main step in the oceans cycling of nutrients that is traditionally attributed mainly to bacteria [34,35,36]. Also, fungi can substantially contribute to chitin degradation and recycle, particularly in some marine environments, such as sediments and estuaries [29,34,37]. However, as mentioned before, these microorganisms have been somehow overlooked with regards to the ability to degrade chitin in marine environments, and consequently less studied for their potential biotechnological applications.

### 2.1. Fungal Growth and Enzyme Activity in Static and Shaken Cultures

**Figure 1:**
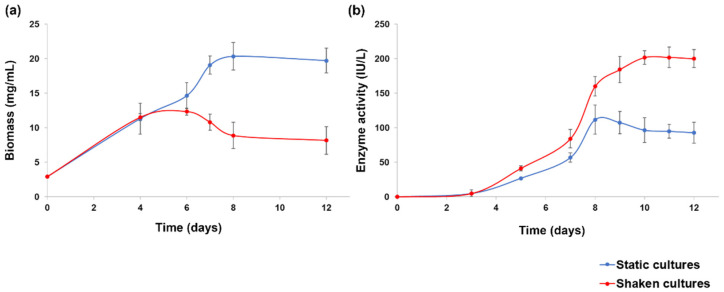
*Clonostachys rosea* IG119 growth (**a**) and enzyme activity (**b**) in static and shaken cultures.

Very scarce literature deals with static liquid cultures for the production of chitinases, which are generally obtained in shaken (or stirred) cultures or in solid-state conditions [38]. In a couple of works, static cultures demonstrated to be more efficient than shaken cultures both for biomass and chitinase production by *Aspergillus terreus* and *Alternaria alternata* [39,40]. However, a preliminary test, to evaluate these different strategies for strain IG119, was planned. The tests demonstrated that static cultural conditions promoted best *C. rosea* IG119 growth (Figure 1a). By contrast, highest enzyme activity was achieved in shaken cultures reaching, after 10 days, the 181% of the maximum observed in static conditions (Figure 1b). Therefore, shaken cultures were selected for the subsequent experiments, also in view of possible better kinetic parameters (productivity and specific production), due to the lower biomass production in relation with the increased enzyme activity.

### 2.2. Growth at Different pHs and Salinities

*C. rosea* IG119 was tested for its growth tolerance at different pHs (pH 4.0, 8.0 and 12.0) and salinities (0, 40, 60, 80, 100, and 120‰) on solid (MEA) and liquid (MEB) modified media to adjust pH and salinity. In general, the fungus was able to grow at all tested pHs (at 0 and 40‰ of salinity) and salinities (at pH 8.0).

**Figure 2:**
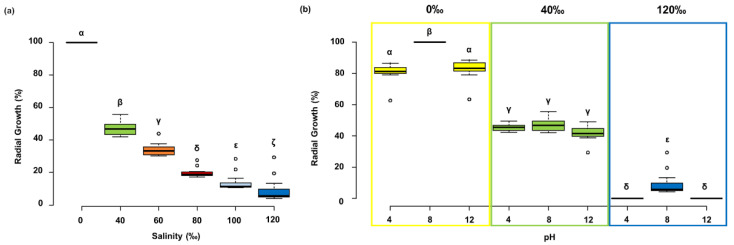
Box plots of radial growth by *Clonostachys rosea* IG119 on solid media. (**a**) Growth at different salinities at pH 8. (**b**) Growth at different pHs and salinities. All data collected daily during the experiment (16 days) were standardized on daily maximum. The same greek letters over the boxes indicate not significant differences among data, as determined by the post hoc test (*p* < 0.005).

Similar growth data were recorded on solid and liquid media (only data on solid media were reported). On solid media the colony radial growth was significantly reduced (*p* < 0.005) proportionally to the salinity increase (Figure 2a). The best fungal growth was obtained in the absence of salt at pH 8.0. Moreover, quite high growth (>80% of the maximum) was observed also at pH 4.0 and 12.0. At salinity 40‰ no significant differences were recorded at all of the tested pH (*p* > 0.005). Finally, at 120‰ the growth was recorded only at pH 8.0 (Figure 2b). Similar data were obtained on liquid media (data not shown). To verify the limits of pH tolerance, pH 2.0 and 3.0 were additionally tested in liquid cultures in absence of salt (optimal condition). The growth was also recorded at pH 3.0.

The ability of some strains of *C. rosea* to grow in a wide pH range is a well-known feature [41,42,43]. In addition, similarly to IG119, some endophytic strains, associated to halophytic plants, showed a growth preference in alkaline conditions [43,44]. Although, some marine strains of *C. rosea* have optimal growth at the sea salinity [44], the broad euryhalinism shown by strain IG119 was never reported before. The available data report the species growth to less than 50‰ of NaCl [41]. Therefore, as far as we know, IG119 is the only *C. rosea* strain able to grow up to 120‰ of salinity.

According to these preliminary results, IG119 growth and activity under polyextremophilic conditions were tested by a RSM factorial design, in the range of salinity 0–120‰ and pH 3.0–12.0.

### 2.3. Biomass and Chitinolytic Enzyme Production in Polyextremophilic Conditions by RSM

In order to maximize the information from our data and optimize the number of tests, the experimental design was performed by RSM. This is one of the most efficient tools to get a performing experimental design. This method is widely used to optimize process parameters in fermentation technology and it has also been used to improve chitinase production by fungi [45,46,47].

In this work, RSM was used to optimize the cultural conditions and analyze the range of tolerance for *C. rosea* IG119 growth and enzyme production in relation to pH and salinity. This was carried out in view of possible applications characterized by variable and/or extreme process conditions, such as those found in the bio-conversion of salty chitin-rich wastes (from sea foods) or in chitin hydrolysis characterized by strong pH increase along the process [45].

Thus, RSM optimization and analysis were performed using an experimental model, considering combinations of these two parameters according to a D-optimal design, as suggested by the software. Data were best fitted by a polynomial quadratic equation, as it can be inferred by the good agreement of experimental data with those estimated by the model. As indicated by the ANOVA table (Table 1), the model revealed high reliability and good statistical performance.

The two responses (biomass and chitinolytic activity), tested by RSM in separated experiments, showed significant regression values (probability ≥ 95%) with no lack of fit. The correlation coefficients (R^2^), indicating the fraction of response variation explained by the model, were very high (0.974 and 0.983 for the biomass and chitinase production, respectively), indicating that the models can explain the 97.4% and 98.3% of the response variability. Moreover, Q^2^ values, indicating the fraction of response variation that can be predicted by the models, were rather good (0.955 and 0.930 for the biomass and chitinase production, respectively). Finally, the high F-values showed that the model terms were quite significant.

**Figure 3:**
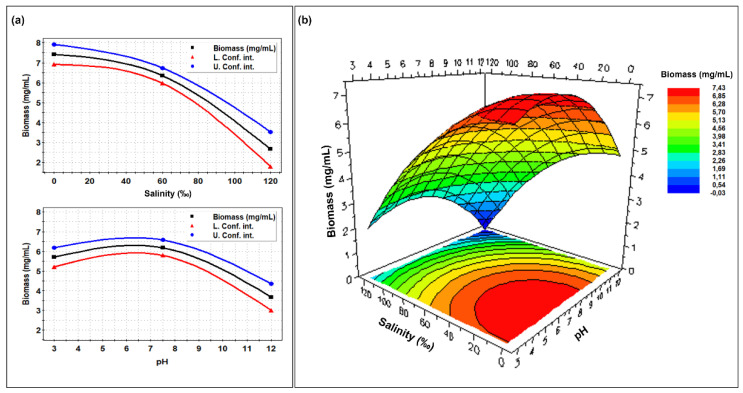
Single (**a**) and combined (**b**) effects of different pHs and salinities on the biomass production by *Clonostachys rosea* IG119 as reported by the RSM model.

**Figure 4:**
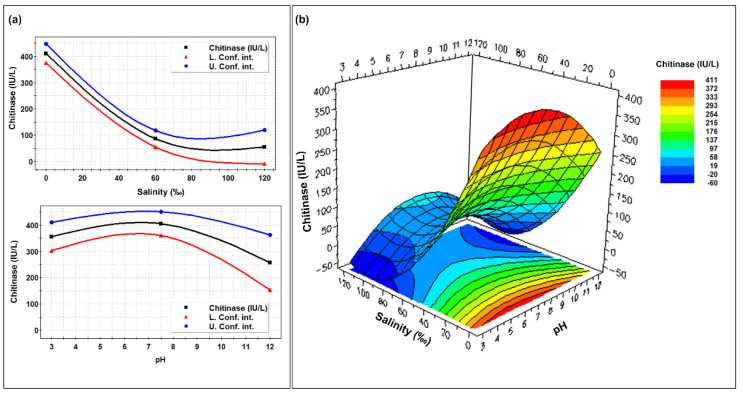
Single (**a**) and combined (**b**) effects of different pHs and salinities on the chitinolytic enzyme production by *Clonostachys rosea* IG119 as reported by the RSM model.

The following plots (Figure 3 and Figure 4) represent the model prediction regarding biomass and enzyme production of the fungus, in relation to pH and salinity, based on the experimental data (see Table 2 in Materials and Methods). The RSM allowed taking into account the effects of the two cultural parameters as considered alone (Figure 3a and Figure 4a) and in combination (Figure 3b and Figure 4b). The strain appeared to be rather halotolerant; salinity started to inhibit the growth after ca. 40‰ and a strong growth reduction (up to 70%) was obtained at 120‰. As for pH, rather good growth was recorded between 3.0 and 8.0, with optimum at ca. pH 6.5. The model indicates highest biomass production (red area of the contour plot, Figure 3b) in the range of pH 3.0–9.0 and 0–40‰ of salinity. However, still significant values of biomass (80% of that in the red area) were predicted in wider ranges (pH 3.0–12.0 and salinity 40–65‰ (Figure 3b)).

The enzyme production appeared to be more affected by salinity than biomass, since it was inversely proportional to NaCl concentration. At ca. 30‰ of salinity it was already 50% of the maximum, to drop at ca. 25% at 60‰ of salinity. Optimal production was predicted at ca. pH 6.5, but very good production (>80% of the maximum) was indicated between pH 3.0 and 10.3 (Figure 4).

By running the software “optimizer” function, the models suggested the following conditions for maximal biomass and enzymes production: pH 6.0 and 6‰ of salinity for biomass, and pH 6.4 and 0‰ for the enzyme production. According to these suggestions a maximum of 7.42 mg/mL (dw) of biomass and of 411.137 IU/L of activity were expected. Biomass and enzyme activity obtained by the experimental validation of the suggested conditions were even higher than the expected values, particularly for the chitinase production that reached 511 ± 18 IU/L at 144 h.

A number of studies investigated the role of *C. rosea* as mycoparasite of phytopathogens; in this context the production of chitinases is always mentioned [41,48]. However, very little is known regarding the production of these enzymes by the fungus in liquid cultures. In addition, specific comparisons are often difficult due to the different methods used to quantify the enzyme activity, which often is not expressed as International Units (IU). For example, the strain of *C. rosea* studied by Chatterton and Punja [49] appeared to be a rather good producer of these enzymes (in liquid cultures), but the authors reported the chitinolytic activity as specific activity (U/g).

The IG119 chitinolytic activity seems to be quite high if compared to that of other fungi, known for their high production of these enzymes (i.e., *Trichoderma* spp., *Penicillium* spp. and *Lecanicillium* spp.). Among them, the mycoparasitic ascomycetes *T. harzianum* and *Lecanicillium* spp. (*L. lecanii* and *L. muscarium*), considered one of the best producers of chitinolytic enzymes, are widely used for pest biocontrol and the commercial production of the enzymes [16,50,51]. Noteworthy, some selected strains of *T. harzianum* (P 1, T 22 and CECT 2413) and *L. muscarium* CCFEE 5003 [16,21], showed same levels of crude enzyme activity (ca. 250 IU/L after 72 h of growth) of *C. rosea* IG119, as reported by Pasqualetti et al. [47]. However, the activity of IG119 increased up to ca. 400 IU/L after RSM optimization of the culture medium [47].

*Thermomyces lanuginosus* ITCC 8895 has been recently mentioned as a “notable” chitinolytic activity producer, the authors reported an activity of 200 IU/L after RSM optimization of culture medium [52,53]. *Duddingtonia flagrans* is one of the most promising fungi studied recently; it was able to produce more than 1000 IU/L, after cultural medium optimization by RSM [54]. Although a more comprehensive comparison with literature would be possible, it is reasonable to state that *C. rosea* IG119 chitinase production is sufficiently high to consider this new marine strain as a promising organism for applications, particularly in case of high concentration of salt and in processes with uncontrolled pH.

To the best of our knowledge, there is no study considering the combined effect of pH and salinity on the chitinase production by fungi, and also the single effect of salinity is scarcely investigated. However, considering that several marine fungi show a general broad euryhalinism [32], their production of chitinase could occur in a wide range of NaCl concentration, although most of the authors limit their investigation to the sea salinity [55]. However, one of the broadest ranges of chitinase production with respect to salinity seems to be that of the marine strain *Beauveria bassiana* BTMF S10, cultivated in solid state, which released its enzymes up to 200‰ NaCl [56].

Generally, maximum fungal chitinase production is observed at acidic or sub-acidic pH [38]. Chatterton and Punja [49] reported that the maximum chitinase activity by a soil strain of *Clonostachys rosea* f. *catenulata* was obtained at pH 6.0, with ca. 30% of reduction at pH 4.0, 5.0, 7.0, and 9.0. As said, strain IG119 maintained more than 80% of its activity when cultivated in the range 3.0–10.0 of pH, with optimum at pH 6.5.

### 2.4. Activity of the Crude Enzyme Extract in Polyextremophilic Conditions

This set of experiments was carried out just to support the polyextremophilic features of the fungus and its total chitinolytic activity. Moreover, the tests were conducted only on the crude enzyme preparations, since in view of applications in real environmental conditions, purified enzymes are generally not used due to their excessive cost. The availability of a crude enzyme preparation, which possibly contains a pool of enzymes with different characteristics, is a further benefit in view of industrial and/or environmental applications (i.e., degradation of chitin-rich materials) for its increased efficiency in chitin hydrolysis. Actually, various commercial preparations are constituted by a mixture of different chitinolytic enzymes [38].

The activity of IG119 crude extract was preliminary screened in two set of experiments, keeping one of the parameters (pH or salinity) constant and varying the other one (pH 2.0–13.0 and salinity 0–120‰). The constant values were pH 8.0 and salinity 38‰, according to the condition found in the natural habitat of the fungus (Tyrrhenian Sea). Subsequently, the activity was tested in polyextremophilic conditions with different combinations of pH and salinity (see Table 3 in Materials and Methods).

**Figure 5:**
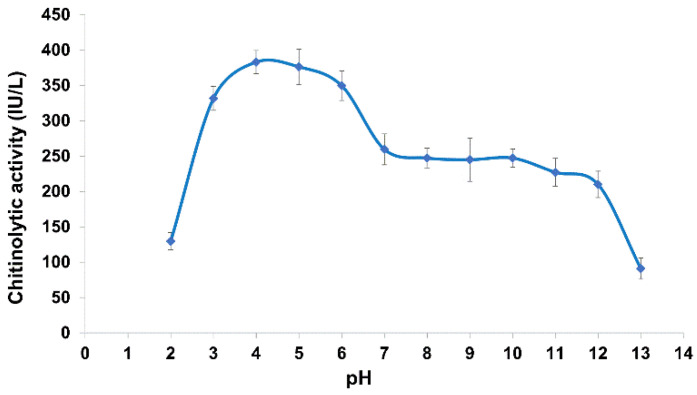
Effect of pH on the Clonostachys rosea IG119 crude enzyme activity.

The activity showed a very peculiar profile in relation to pH (Figure 5). It increased from pH 2.0 to pH 4.0 (up to 383 IU/L) then markedly declined thereafter until pH 7.0, where it dropped to 65% of the maximum. According to the typical enzyme kinetics, in relation to chemical/physical parameter variations, a further strong activity reduction would be expected with further pH increase. Surprisingly, the activity remained constant in broad alkaline conditions up to pH 12.0 and finally declined thereafter.

This peculiar profile suggests possible presence of diverse chitinolytic enzyme sets which are active in different pH conditions. The activity observed at the extreme pH values (130 IU/L and 91 IU/L at pH 2.0 and pH 13.0, respectively) indicates a great ability of the crude enzyme to act as pool of enzymes with a very broad activity range (Figure 5). No significant differences were recorded for the enzyme activity tested at pH 8.0 for different salinities, indicating a substantial stability of the crude enzyme in relation to this parameter (data not shown).

**Figure 6:**
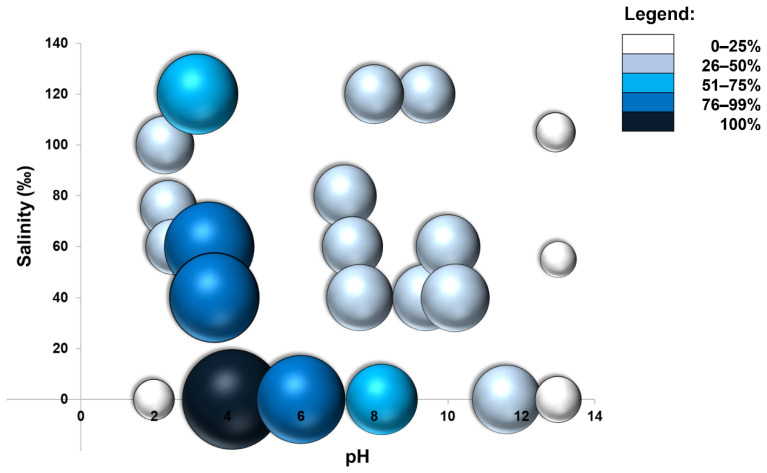
Activity of *Clonostachys rosea* IG119 crude extract in polyextremophilic conditions.

The crude enzyme was active also in polyextremophilic conditions. As expected, maximum activity was registered in absence of salt and pH 4.0. Nevertheless, the activity was registered in all tested conditions and it was relatively high in the pH range 3.0–6.0 at all of the salinities tested. From neutral to strong alkaline conditions (pH 7.0–12.0), a reduction of activity was observed. However, high values were still recorded (i.e., 156 IU/L at pH 12.0 and 0‰ of salinity). Noteworthy, also at the most extreme conditions tested (pH 2.0–3.0 and pH 13.0 and salinity 100–120‰) the crude enzyme maintained a rather good activity, particularly in acidic conditions (Figure 6).

As mentioned above for the enzyme production, no literature is available regarding the activity of crude or purified enzymes with regard of combinations of salinity and pH. However, effect of pH on the activity of chitinolytic enzymes is generally more studied than that of salinity. Chung et al. [30] studied the separate effects of pH and salinity for the crude enzyme by a marine derived strain of *Acremonium* sp., showing that its activity was recorded in between pH 4.6 and 9.0 (optimum at 6.0–7.6 pH) and salinity in the range 0–50‰ with optimum at 15‰.

Considering pH, the reported activity ranges of chitinase crude extracts are generally more limited than that of strain IG119. The enzyme from *Aspergillus niger* studied by Brzezinska and Jankiewicz [57] was active in the range of 4.0–7.5 pH, with an optimum pH of 6.0. *Trichoderma virens* crude enzyme showed optimal activity at pH 3.0; at pH 2.0 and 5.0 its activity was ca. 60% less than the optimum, being ca. 90% less than the optimum at pH 10.0 [58]. Although the activity range of this enzyme was quite similar to that of IG119, its reduction, at values different from the optimum, was definitely much more consistent. Similar trends were recorded for *Penicillium aculeatum* NRRL 2129 and *Metarhizium anisopliae* ME 1, but even with much more narrow ranges [59,60].

## 3. Materials and Methods

### 3.1. Microorganism

The strain IG119 was isolated in the Tyrrhenian Sea from the marine phanerogam *Posidonia oceanica* and cryogenically maintained at −40 °C in the culture collection of microorganisms of the “Laboratorio di Ecologia dei Funghi Marini”, DEB (University of Tuscia, Viterbo, Italy). The strain was identified as *Clonostachys rosea* in a previous work through a polyphasic approach, combining morphological (colony structure and morphology on different media, and microscopic observations of reproductive structures), physiological (growth at different pH, salinity and temperature), and molecular (taxonomic inference based on ITS region sequences) methods [32]. The strain has been revitalized and sub-cultured on modified Malt Extract Agar: 50 g/L (MEA, Sigma-Aldrich, St. Louis, MO, USA) in distilled water added with Sea Salts (Sigma-Aldrich, St. Louis, MO, USA) to a final concentration of 38‰.

### 3.2. Fungal Growth and Enzyme Activity in Liquid Static and Shaken Cultures

The strain was tested for its optimal growth and enzyme production both in liquid static and shaken conditions. Liquid cultures were carried out on Malt Extract Broth (MEB, Difco, Detroit, MI, USA) and on an Inducing Medium (IM) containing 1% of colloidal chitin and 0.5% of Yeast Nitrogen Base (YNB, Difco, Detroit, MI, USA) [21]. Colloidal chitin was prepared from crab shells chitin (Heppe Medical Chitosan GMBH, Halle, Germany) following the protocol reported by Fenice et al. [61]. All media were modified adding Sea Salts to a final concentration of 38‰ and autoclaved at 121 °C for 20 min.

Erlenmeyer flasks (500 mL) containing 100 mL of medium were inoculated with ca. 2.5 mg/mL dry weight (dw) of mycelium grown for 5 days on MEB, and incubated at 28 °C for 12 days in a thermostatic chamber (static condition) and in an orbital shaker at 150 rpm (shaken cultures).

The growth was estimated daily on MEB cultures by dw determinations: one Erlenmeyer flask culture was filtered to separate the broth from the biomass and dw was obtained with a thermobalance (Eurotherm, Gibertini Elettronica, Novate Milanese, Italy). Samples for chitinolytic activities were withdrawn daily from cultures in the IM. The experiments were carried out in triplicate.

### 3.3. Growth Tolerance at Different pHs and Salinities

The selected strain was tested in solid and liquid culture for its growth tolerance at different pHs (pH 4.0, 8.0, and 12.0) and salinities (0, 40, 60, 80, 100, and 120‰), using MEA and MEB media modified to adjust pH and salinity.

Pre-poured plates (90 mm diameter) were inoculated with a punctiform inoculum obtained using sterile needles from 5/7-day cultures grown on MEA slants (at 28 °C). Erlenmeyer flasks (500 mL) containing 100 mL of medium were inoculated as previously reported.

Plates were incubated at 28 °C and growth diameter was measured daily for 16 days. The liquid cultures were incubated in an orbital shaker (28 °C; 150 rpm) and the growth (dw) was estimated at the 16th day. Three replicates were performed for each analysis.

### 3.4. Biomass Production and Chitinolytic Activity in Polyextremophilic Conditions by RSM Factorial Design

Effect of the combined action of salinity and pH on the biomass and chitinolytic activity was optimized by a D-optimal design, with the following independent variables (factors):

$$X_{1}=\mathrm{salinity}\;\left(‰\right)$$

$$X_{2}=\mathrm{pH}$$

The above dimensional independent variables were coded as dimensionless terms by the following equation:

$$X_{i}=\left(A_{i}-A_{0}\right)/\Delta A;\;i=1,2$$

where X*_i_* is a coded value and A*_i_* is the actual value of the variable, A_0_ is the actual value of the same variable at the center point and ΔA is the variable step change.

The range of the variables is given in Table 2.

**Table 2:** Experimental setup combining different salinities and pHs as suggested by the model.

Experiment	pH	Salinity (‰)
N1, N17	3.0	0
N2, N18	12.0	0
N3, N19	3.0	120
N4, N20	12.0	120
N5	3.0	60
N6	12.0	60
N7	7.5	0
N8	7.5	120
N9, N13	7.5	40
N10, N11	7.5	60
N12	10.0	40
N14	3.0	40
N15	7.5	80
N16	12.0	40

Data were subjected to analysis of variance (ANOVA) and fitted according to a second-order polynomial model shown by:

$$Y={\;\beta }_{0}+\sum \beta_{i}X_{i}+\sum \beta_{\mathrm{ii}}X_{i}^{2}+\sum \beta_{\mathrm{ij}}X_{i}X_{j}$$

where Y is the predicted response variable, β_o_ is the intercept, β_i_ and β_ii_ linear coefficient and quadratic coefficient, respectively, β_ij_ is the interaction coefficient, and X_i_ and X_j_ are the coded forms of the input variables. To estimate the impact of single independent variables on the response, main effects were calculated using:

$$Y={\;\beta }_{0}+\beta_{i}X_{i}+\beta_{\mathrm{ii}}X_{i}^{2}$$

Statistical examination of results and response surface study were carried out by the MODDE 5.0 software (Umetrics AB, Umeå, Sweden).

Fermentations under optimal conditions for best biomass and enzyme production, as suggested by the model, were carried out in triplicate in subsequent experiments. All media for the RSM optimization were inoculated as mentioned above.

The experiment for biomass was carried out for 10 days and activity was measured a 144 h.

### 3.5. Activity of the Crude Extract in Polyextremophilic Conditions

The crude extract, obtained in optimal growth conditions, was tested for its activity at different pHs (range 2.0–13.0, steps of 1.0 pH), and salinities (range 0–120‰, steps of 20‰); these factors were studied alone and in combination. The activity at different pHs was tested at 38‰ of salinity; while salinity was tested at pH 8.0. In addition, the activity was tested also in polyextremophilic conditions of salinity and pH (Table 3) using different 0.1 M buffer solutions (pH 2.0–8.0: Citrate Phosphate Buffer (McIlvaine Buffer); pH 9.0 Carbonate-Bicarbonate Buffer; pH 10.0: NaHCO_3_/NaOH Buffer; pH 11.0–12.0: Na_2_HPO_4_/NaOH Buffer; pH 13: KCl/NaOH Buffer) and appropriate calibration curves. The pH was measured for each saline solution.

**Table 3:** Experimental setup combining different salinities and pHs.

Experiment	pH	Salinity (‰)	Experiment	pH	Salinity (‰)
N1	9.4	120	N12	3.18	0
N2	10.0	60	N13	3.5	0
N3	10.2	40	N14	3.64	120
N4	11.6	0	N15	4.13	120
N5	9.4	60	N16	2.0	60
N6	8.0	60	N17	13.3	60
N7	7.2	0	N18	12.93	0
N8	7.4	120	N19	13.01	120
N9	7.6	40	N20	2.3	40
N10	8.2	60	N21	2.4	60
N11	6.0	40	N22	2.52	40

### 3.6. Analytical Methods

For the secondary screening and RSM optimization the overall chitinolytic activity (expressed in International Units, IU) was determined by the method of Dinitrosalicylic Acid (DNSA), using N-Acetyl-D-glucosamine (NAG) for standard curve, as previously reported [61,62].

One unit of enzyme activity was defined as the amount of enzyme which released 1 µmol of NAG per mL per min under the assay conditions. For the enzyme assay, suspensions of colloidal chitin in appropriate buffers were used, as mentioned above.

In order to avoid interferences by different salinities and pHs, specific calibration curves were made for each tested condition.

### 3.7. Data Analysis

One-way ANOVA and post hoc tests (Tukey) were performed to compare strain growth and activity (SYSTAT v. 8.0, SPSS Inc. Chicago, IL, USA 1999); *p*-value ≤ 0.05 was considered statistically significant. In addition, non-parametric tests (Kruskal–Wallis, one-way analysis of variance, and Kolmogorov–Smirnov two-sample tests) were performed on preliminary data assessments obtained by Shapiro–Wilk (normal distribution) and Levene tests (homogeneity of variance). All of these tests were run using Systat 8.0.

## 4. Conclusions

*Clonostachys rosea* IG119 was able to grow and release high levels of chitinolytic enzymes in a very broad range of pH and salinity. In addition, its crude enzyme solution showed high activity in a broader range of conditions, and therefore it was able to efficiently catalyze the chitin hydrolysis also in severe polyextremophilic conditions.

All of these features indicate that this promising marine fungus and its chitinolytic enzymes could be considered as new resources for biotechnology. This suggests possible uses in applications with variable conditions of pH and salinity, such as the valorization and treatment of chitin-rich salty wastes from seafood industry. Moreover, since chitin degradation produce significant pH increase, the use of the versatile IG119 crude enzyme could reduce the process costs related to the pH control. The RSM optimization carried out in this study permitted to increase the enzyme production of ca. 150% (from the initial value of ca. 200 IU/L up to 511 IU/L). Nevertheless, the strain IG119 has not been tested in bioreactor yet. The cultivation under controlled optimized conditions in a bioreactor would permit further increase of process yield and productivity. Although not particularly important for possible environmental/industrial applications, purification and characterization of the chitinolytic enzymes produced by *Clonostachys rosea* IG119 could be necessary to obtain a focus on its catalytic features.

## Figures and Tables

**Table 1:** Coefficients of the input parameters (estimated coefficient, standard error and significance) and ANOVA table showing statistical parameters measuring the correlation and significance of the models.

	Biomass (mg/mL)			Activity (U/L)		
Coefficient	RC	SE	P	RC	SE	P
Constant	6.3549	0.1772	3.55 × 10^−15^	120.434	12.9709	1.47 × 10^−5^
pH	−0.0163	0.2381	0.9463	40.0609	17.9274	0.0559
Salinity	−2.3774	0.2031	1.28 × 10^−8^	−188.5	11.5727	2.03 × 10^−7^
pH*pH	−2.6611	0.4219	1.93 × 10^−5^	−77.5963	16.6379	1.62 × 10^−3^
Sal*Sal	−1.3177	0.2678	0.0002	−101.544	13.1646	5.67 × 10^−5^
pH*Sal	−0.0168	0.2654	0.95043	25.7179	23.5271	0.3063
**ANOVA table**						
F value	103.227	*p* = 0.000		93.59	*p* = 0.000	
Q^2^	0.955			0.930		
R^2^	0.974			0.983		
R^2^-adjusted	0.964			0.973		

Legend: RC, regression coefficient; SE, standard error; *p*, p-value.

## Data Availability

The data presented in this study are available on request from the corresponding author.

## Sample Availability

Samples of the microbial strain are available from the authors.
